# Obstetric vesico-uterine fistula in nine reference hospitals in the Democratic Republic of the Congo: epidemiological, clinical, and therapeutic aspects

**DOI:** 10.1186/s12905-024-03124-w

**Published:** 2024-05-23

**Authors:** Justin Lussy Paluku, Cathy Mufungizi Furaha, Susan A. Bartels, Barthelemy Kasi Aksanti, Benjamin Kasereka Kataliko, Jonathan ML Kasereka, Eugénie Mukekulu Kamabu, Benjamin Kambale Kalole, John Kasereka Muteke, Michel Mulyumba Kyembwa, Richard Kabuseba Kabuyanga, Zacharie Kibendelwa Tsongo, Stanis Okitotsho Wembonyama, Charles Wembonyama Mpoy, Jeannot Sihalikyolo Juakali

**Affiliations:** 1 Department of Obstetrics and Gynecology, Faculty of Medicine, University of Goma, Goma, Democratic Republic of the Congo; 2 Department of Obstetrics and Gynecology, HEAL Africa Hospital, Goma, DRC Democratic Republic of the Congo; 3 Departments of Emergency Medicine and Public Health Sciences, Queen’s University, Kingston, Canada; 4 Department of Orthopedics and Trauma, HEAL Africa Hospital, Goma, DRC Democratic Republic of the Congo; 5 Department of Internal Medicine, HEAL Africa Hospital, Goma, DRC Democratic Republic of the Congo; 6 Department of Internal Medicine, Faculty of Medicine, University of Kisangani, Kisangani, DRC Democratic Republic of the Congo; 7 Departments of Pediatrics and Public Health, Faculty of Medicine, University of Lubumbashi, Lubumbashi, DRC Democratic Republic of the Congo; 8 Department of Obstetrics and Gynecology, Faculty of Medicine, University of Lubumbashi, Lubumbashi, DRC Democratic Republic of the Congo; 9 Department of Obstetrics and Gynecology, Faculty of Medicine, University of Kisangani, Kisangani, DRC Democratic Republic of the Congo

**Keywords:** Obstetric vesico-uterine fistula, Cesarean section, Democratic Republic of the Congo

## Abstract

**Introduction:**

With global cesarean section rates rising, there’s concern about increase in obstetric vesico-uterine fistula (OVUF). Very little is known about this anatomoclinical entity of obstetric fistula in Africa in general and in DRC in particular. Our purpose was to describe the epidemiological, clinical, and therapeutic aspects of OVUF in the Democratic Republic of the Congo (DRC).

**Methods:**

This was a descriptive cross-sectional study. Data were collected from patients who presented with OVUF across seven provinces of the DRC (North Kivu, Haut-Uélé, Kasai Central, Kwilu, Maniema, Nord-Ubangi and Sankuru) from January 2017 to December 2022. Study variables were epidemiological, clinical, and therapeutic features.

**Results:**

Of 1,267 patients presenting with obstetric fistulas, 355 (28.0%) had OVUF. The mean age was 32.9 ± 11.6 years, 80.6% of patients (286/355) lived in rural areas, and the majority had a low level of education (40% no formal education, 30.1% primary school, 28.7% secondary school). In total, 64.8% of patients were primiparous (230/355) and in all (100%) cases, OVUF was caused iatrogenically during cesarean delivery. Majority (76.3%) of patients laboured for one day or less (mean duration 1.0 ± 0.5 days) before giving birth, and the fetus died in 58.3% of cases. In 35.8% of cases, the fistula had lasted more than 10 years (mean age 10.1 ± 10.0 years) before repair. A proportion of 88.2% (*n* = 313) of OVUF was isolated while 11.3% (*n* = 40) was associated with a uretero-vaginal fistula. In 82.8% (*n* = 294) of cases the OVUF was single. The average fistula size was 2.4 ± 1.0 cm (range: 0.5 and 5.5 cm) and 274 (77.2%) fistulas measured between 1.5 and 3 cm, with 14.9% (*n* = 53) of them larger than 3 cm. Fibrosis was present in 65.1% of cases, cervical involvement was absent in 97.7% and post-operative complications were absent in 94.4%. In all cases, the OVUF was surgically repaired abdominally with a success rate of 97.5% (346/355).

**Conclusion:**

The proportion of OVUF is relatively high in the DRC. Most affected patients were young, under-educated, primiparous women living in rural areas. Cesarean section was the sole identified cause of OVUF which was isolated, single, without fibrosis, in majority of cases. Abdominal repair of OVUF was very effective, with good results in almost all cases. Teaching young doctors working mainly in remote areas how to perform safe cesarean section is needed to reduce incidence of OVUF in DRC.

## Introduction

Female urogenital fistulas are pathological communications between the urinary tract and the genital organs. Among these fistulas is the vesico-uterine fistula, an anatomoclinical entity in which the bladder communicates with the uterus. The most frequent causes of urogenital fistulas are obstetrical or surgical. Also, they can be caused by tumors or radiation necrosis [1, 2]. In the literature on obstetric fistulas in the DRC, vesico-vaginal fistulas are the most common anatomoclinical entity of urogenital fistulas [3].

Obstetric vesico-uterine fistulas (OVUF) are less common, accounting for 2–9% of all urogenital fistulas [4–6]. Nsambi et al. [7], in a series of 413 genitourinary fistulas collected in Katanga province (Democratic Republic of the Congo), reported 92.3% vesico-vaginal fistulas, 5.6% recto-vesico-vaginal fistulas, 1.2% recto-vaginal fistulas, 0.5% uretero-vaginal fistulas and 0.5% vesico-uterine fistulas. However, in another study carried out in Bukavu, in the eastern DRC [8], the proportion of vesico-uterine fistulas was 11% in the group of fistula patients who had delivered by cesarean section.

Given the continuing increase in cesarean section rates worldwide, it is likely that the relative frequency of OVUFs will continue to increase and be revised upwards [8] from that reported in the current literature. In regions where hospital deliveries are the rule, an increase in the frequency of OVUF secondary to surgical interventions, such as cesarean sections, cesarean hysterectomies and others, has been observed [9].

In fact, the leading cause of OVUF is iatrogenic injury during cesarean section, especially during dissection of the lower segment of the uterus, which accounts for 83–93% of all OVUF cases [10–13]. At the time of hysterorraphy, the dome of the bladder can be incorporated into the uterine incision, particularly when a low-segment cesarean section is performed at full cervical dilatation. There is also a risk of inadvertent opening of the bladder during such a cesarean section, which remains unrecognized. Less commonly, rare obstetric complications such as rupture of the uterus during vaginal delivery can be the cause, particularly after a previous cesarean section or with the use of forceps [14–16], uterine curettage, manual removal of the placenta, placenta praevia [16]. Therefore, it appears that OVUFs are high fistulas of iatrogenic origin.

The aim of the present study was to describe the sociodemographic, obstetric, clinical and therapeutic characteristics of patients with OVUF in the DRC.

## Materials and methods

We conducted a descriptive cross-sectional study. A total of 355 patients who presented with OVUFs from January 2017 to December 2022 after community sensitization campaigns were enrolled. The sensitization campaigns, which included raising community awareness about obstetric fistulas, were organized by the non-governmental organization HEAL Africa in collaboration with the Congolese National Ministry of Public Health with the goal of improving access to specialized care. Seven provinces in the DRC were included: North Kivu, Haut-Uélé, Kasai Central, Kwilu, Maniema, Nord-Ubangi and Sankuru. Patients with OVUF were enrolled both from outpatient clinics and from referrals for surgical management during the study period and in the study setting. This was particularly the case for the HEAL Africa Hospital in Goma, North Kivu province, which routinely treats fistula patients.

A total of 18 mobile surgery campaigns were carried out in 9 reference hospitals in 7 provinces of the DRC. In 2 of the 7 provinces (Maniema, Nord-Kivu), the campaigns took place in 2 hospitals each.

All the patients in this study were examined and treated by the same team led by a gynecologist and fistula surgeon who worked as the main surgeon. The team also included a nurse anesthetist who oversaw coordination of post-operative care. The latter had extensive experience in urogynecological nursing. There was also an experienced nurse serving as an operating room technician. He was responsible for coordinating activities in the operating room. Equipment used were the same for all patients. Thus, all patients were repaired according to the same standards of care and were subjected to the same therapeutic protocol.

Any patient with history of uncontrolled loss of urine after caesarean section and whose vaginal examination failed to reveal a fistula, was suspected to have a high fistula. Thus, an examination under anesthesia (EUA) with a methylene blue test (Dye test) was performed to try to locate the fistula. This examination took place in the operating room under spinal anesthesia, with proper lighting and good perineal muscle relaxation. All the patients presented in this study had high fistulas, which were either not visualized on EUA because too high or were only partially visualized under traction of the cervix. Confirmation of fistula was obtained by outflow of methylene blue through the cervix on dye test. The dye test consisted of instillation of diluted methylene blue solution in the bladder through a catheter with a Sims speculum inserted in the vagina. Methylene blue solution was then visualized, coming from the cervix. All surgical repairs carried out on these patients revealed high fistulas (vesico-uterine fistulas). Fistulas were measured during the surgical repair.

Patients underwent surgical repair according to the same protocol. In the operating theatre, after spinal anesthesia was given, patient was put in supine position, and a bladder catheter was aseptically inserted. Aseptic cleaning of abdomen and draping were done. Through a midline suprapubic incision, the Retzius space was opened. From this space, the bladder was identified and mobilized on its anterior surface. A cystostomy was performed to help identify the fistula. Ureters were then identified and catheterized, before the fistula was repaired. Fistula repair consisted of the following steps: Infiltration of the vesico-uterine wall with hemostatic solution (mixture of Adrenaline, Lidocaine, Normal saline), incision made around the fistula, dissection of the vesico-uterine wall for bladder mobilization, suture of the uterine wall in one layer, repair of the fistula in two layers, cystorraphy in two layers. This was abdominal “extraperitoneal trans-vesical” repair of the vesico-uterine fistula (VUF). When local conditions did not allow easy access and adequate mobilization of the anterior surface of the bladder, the peritoneum was opened. Vesico-uterine dissection was done to separate the bladder from the uterus with the aim of gaining access to the fistula and exposing it fully. For better fistula repair, a cystostomy was performed to identify and catheterize the ureters. Fistula margins on the uterine wall were revived then closed in one layer. Fistula on the bladder was repaired in two layers and a two-layer cystorraphy was performed. This was abdominal “transperitoneal transvesical” repair.

Ureters that would not be catheterized, were assumed to be blocked or to have ureteric fistulas, then uretero-vesical reimplantation was performed.

Data were collected from patients at the time of history taking, physical examination, surgical repair and end of post-operative follow up (hospital discharge).

Data were double-entered in a computer on two Excel sheets. Data were then pooled, errors were corrected and any missing information completed. A single database was created and transferred into STATA 16 software for statistical analysis.

We investigated the sociodemographic and obstetric characteristics of patients with OVUF, as well as the fistula-related parameters, and therapeutic aspects. Statistical analyses were conducted using the STATA 16 software to describe the epidemiological, obstetrical, clinical, and therapeutic characteristics of OVUF with frequencies (%) and means (with standard deviation).

The study was approved by the Medical Ethics Committee of the University of Goma (Approval No.: UNIGOM/CEM/011/2022). The data was collected anonymously. The study did not present any direct monetary benefits for the study participants.

## Results

**Fig. 1:**
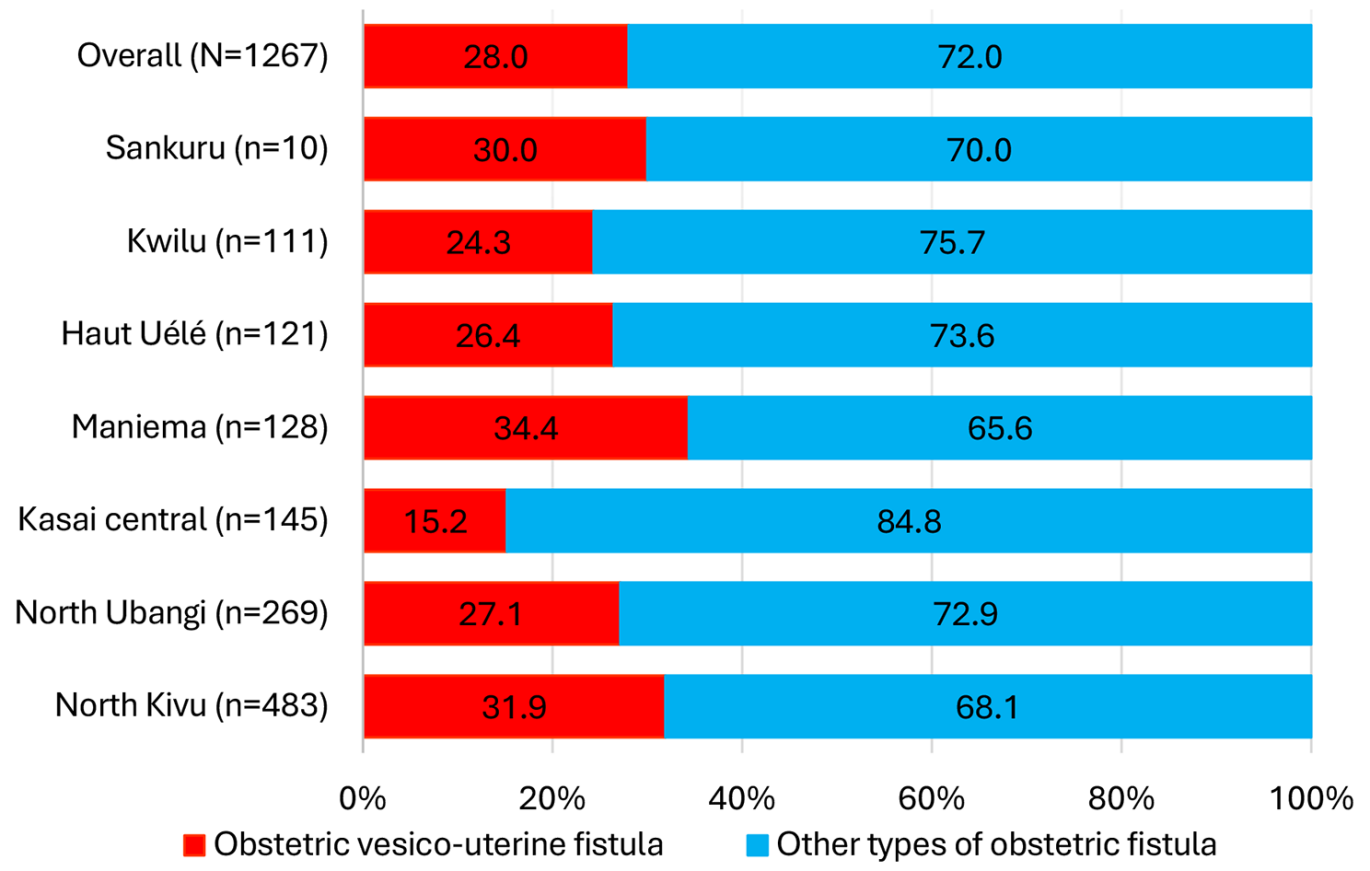
Distribution of cases of OVUF among all cases of obstetric fistula recorded in the seven provinces

During the study period, we treated 1,267 patients with obstetric fistulas among whom 355 had OVUF (28.0%). Figure 1 shows the distribution of cases of OVUF among all cases of obstetric fistula recorded in the seven provinces. Maniema province led with 34.4% (44/128) of cases of OVUF, followed by North Kivu province (31.9%, 154/483). Kasai Central province had the fewest cases of OVUF (15.2%, 22/145).

Table 1 provides the sociodemographic characteristics of included OVUF patients. Among them, 80.6% resided in rural areas. The mean patient age was 32.9 ± 11.6 years and 31.8% of patients were aged 20 to 29 years. Patients had a mean parity of 1.7 ± 1.3 and 64.8% were primiparous. Nearly 40% of patients had no formal education, 30.1% had primary education, and 28.7% had secondary education. Most patients were married (45.9%) with 38.3% being separated or divorced (Table 1).

Patients’ obstetrical history is provided in Table 2. In 76.3% of patients, labour lasted one day or less (mean duration 1.0 ± 0.5 days). A total of 207 (58.3%) patients reported that the fetus did not survive the delivery associated with fistula development.

Table 3 highlights the clinical characteristics of OVUF. Most were isolated fistulas (88.2%) while others were in combination with other fistula types (11.8%). All OVUFs had developed following a cesarean section. The mean age of fistula was 10.1 ± 10.0 years and 35.8% of fistulas were over 10 years old. Majority of patients had a single OVUF (82.8%) and 41.1% had previously undergone at least one surgical fistula repair. The average fistula size was 2.4 ± 1.0 cm (range: 0.5–5.5 cm) and 77.2% measured between 1.5 and 3 cm, with14.9% of fistulas being larger than 3 cm. As for the clinical features of the fistulas, fibrosis was present in 65.1%, cervical involvement was absent in 97.7% and post-operative complications were absent 94.4% of cases. All 355 OVUFs abdominal surgical repair, of which 346 (97.5%) were successful.

**Table 1:** Sociodemographic characteristics of patients with OVUF at the time of surgical repair

Variable	Frequency (*n* = 355)	Percentage
**Residence**		
Urban	69	19.4
Rural	286	80.6
**Age (years)**		
< 20	38	10.7
20–29	113	31.8
30–39	110	31.0
≥ 40	94	26.5
Mean ± standard deviation (range)	32.9 ± 11.6	(16–77)
**Level of Education**		
None	141	39.7
Primary	107	30.1
Secondary	102	28.7
Post-secondary	5	1.4
**Marital status**		
Married	163	45.9
Divorced/Separated	136	38.3
Single	41	11.6
Widow	15	4.2

**Table 2:** Obstetric history of the 355 patients with OVUF

Variable	Frequency (*n* = 355)	Percentage
**Parity at fistula repair**		
1	230	64.8
2–4	103	29.0
≥ 5	22	6.2
**Duration of labour of index delivery (days)**		
≤ 1	271	76.3
	78	22.0
≥ 3	6	1.7
Mean ± standard deviation (range)	1.0 ± 0.5	(0.5–4.5)
**Neonatal outcome of index pregnancy**		
Death	207	58.3
Survival	148	41.7

**Table 3:** OVUF characteristics and surgical repair outcomes

Variable	Frequency (*n* = 355)	Percentage
**Fistula age (years)**		
< 1	60	16.9
1–5	98	27.6
6–10	70	19.7
> 10	127	35.8
Mean ± standard deviation (range)	10.1 ± 10.0	(0.3–45)
**Previous surgical repair**		
0	209	58.9
1	94	26.5
≥ 2	52	14.6
**Association with other types of fistula**		
No associated fistula	313	88.2
Uretero-vaginal	40	11.3
Recto-vaginal	2	0.5
**Number of fistulas**		
1	294	82.8
≥ 2	61	17.2
**Fistula size (cm)**		
< 1.5	28	7.9
1.5-3	274	77.2
> 3	53	14.9
Mean ± standard deviation (range)	2.4 ± 1.0	(0.5–5.5)
**Presence of fibrosis**		
Absent	124	34.9
Present	231	65.1
**Cervical involvement**		
Absent	347	97.7
Present	8	2.3
**Post-operative complications**		
Absent	335	94.4
Hemorrhage	19	5.3
Infection	1	0.3
**Result of surgical repair**		
Success	346	97.5
Failed	9	2.5
**Route of repair**		
Extraperitoneal transvesical	339	95.5
Transperitoneal transvesical	16	4.5

## Discussion

The present study reports that 28% of obstetric fistulas were vesico-uterine. In two Congolese (DRC) studies carried out in Katanga [7] and South Kivu [8] provinces, the proportions of OVUFs were 0.5% and 5.9%, respectively. In a Polish study by Jóźwik et al. [13], 13.6% of 110 recorded genitourinary fistulas were vesico-uterine. In a cohort of 170 urogenital fistulas collected in Burkina Faso by Kaboré et al. [17], 8.2% were vesico-uterine. This frequency was 14.29% in a series of 140 urogenital fistulas reported by Konan et al. [18] in Abidjan (Côte d’Ivoire). Currently, due to the continuing increase in cesarean section rates worldwide [19] and in the DRC in particular [20], the frequency of obstetric vesico-uterine (OVUF) is likely to increase.

The present study shows that the majority (80.6%) of patients with OVUF resided in rural areas. This may have been a contributing factor to the occurrence of OVUF. Healthcare facilities in rural sub-Saharan areas are characterized by a shortage of qualified health care providers and medical inputs [19–21]. This is explained by the fact that most qualified doctors prefer to live and practice in urban areas where they are well paid, and that most rural hospitals have insufficient infrastructure for emergency surgical care [22]. Access to cesarean section is not easy in rural areas, and when women are able to access it, they are sometimes operated on by doctors without sufficient surgical experience and with inadequate equipment. Performing a safe cesarean section requires well-trained and experienced doctors [22]. However, we found that all the hospitals in which we carried out OVUF surgical repairs lacked specialist doctors, surgeons, and obstetrician-gynecologists. Furthermore, the surrounding healthcare facilities were run by nurses who worked alone without appropriate surgical supervision, often in deplorable conditions.

The mean age of patients in the present study was 32.9 years (extremes: 16–77 years) at the time of management. This is high compared with the ages reported by Hadzi-Djokic et al. [5] and Rajamaheswari and Chhikara [23], which were 27 and 30.1 years, respectively. This average age at the time of management appears to be more advanced, given that patients with obstetric fistula have always been unanimously young in several studies carried out in Africa, and that there is an association between young age and feto-maternal disproportionality, which is implicated in the development of obstetric fistulas [24, 25]. This observation is likely explained by the average duration of OVUF in our data, which was 10.1 years (extremes: 0.3–45 years). Overall, results suggest that many patients developed OVUFs at a young age but experienced significant delays in receiving care. In the DRC, most of fistula patients are in remote areas where resources are insufficient. Medical facilities are not well equipped and are poorly staffed with skilled medical personnel. The few specialists who can care for fistula patients are in urban areas. This is why fistula patients spend years before accessing surgical repair [25]. In addition, the DRC lacks adequate infrastructure (roads, means of transport, etc.), especially in rural areas [25], where 52% of the population live [26].

In this series, in 76.3% of cases, the duration of labor was less than 24 h and neonatal outcome was fatal in more than half the cases (58.3%). Even though we did not record it, we presume deliveries that resulted in all these neonatal deaths were emergency cesarean sections. This assumption is in line with the literature, which grants urgent cesarean sections a high risk of major complications [8].

Diagnosis of a VUF is not difficult but is often delayed in our conditions. Diagnosis is usually made by vaginal examination, cystoscopy, cystography and hysterography, or by the use of more advanced methods such as ultrasonography, computed tomography and magnetic resonance imaging [5]. In our series, diagnosis of VUF was made during an EUA using the methylene blue test (Dye test) which is easy to perform, less expensive and accessible even in rural areas.

Rarely, OVUF presents as a triad of symptoms known as Youssef’s syndrome. Clinically, this syndrome is characterized by the association of three signs: cyclic hematuria or menuria, amenorrhea and urinary continence. In this particular entity of VUFs, where the fistulous tract is trans-isthmic, there is a uterine cervico-isthmic sphincter which makes the fistula function only in the uterus-bladder direction [27]. In some cases, OVUF may manifest as watery vaginal discharge or the absence of these symptoms [28]. In the present study, all patients with OVUF presented with the complaint of uncontrolled leakage of urine after cesarean section.

A VUF is an uncommon anatomoclinical form of urogenital fistula. Several decades ago, instrumented vaginal deliveries (forceps, vacuum, …) [29–31] were the predominant cause of OVUFs. Recent studies [5, 23, 32] including ours, now indicate cesarean section, specifically on the lower uterine segment, as the prevailing etiology. The increase in the number of cases of OVUFs is probably linked to the rise in the number of cesarean deliveries in recent years. As several authors have pointed out [5, 23, 32, 33], the development of these fistulas can be explained by occult or overt bladder lesions during cesarean delivery. Inadequate mobilization of the bladder or direct bladder injury adherent to the lower segment are factors responsible for the bladder injuries most frequently encountered during cesarean Sect. [23]. Other factors may also account for bladder injury during cesarean section, such as clamping injury, extensive dissection, diathermic cauterization or even inclusion of the bladder wall during uterine closure [8, 27, 34]. Although we have not had cases of OVUF after vaginal delivery, it should be noted that other authors [5, 23, 35, 36] had reported the development of OVUF after vaginal delivery in patients who had previously undergone cesarean section. Thinning of the lower uterine segment after vaginal delivery, combined with potential adhesion between the bladder and uterine scar due to previous cesarean section, is likely to initiate and accelerate fistula formation [37, 38].

All our patients were managed surgically, via the abdominal approach. The success rate after surgical repair was 97.5%. In the literature [23, 39–41], several conservative treatment options have been proposed, such as long-term bladder catheterization, hormone therapy and cystoscopic fulguration of the VUF. However, these conservative methods are only effective in 5% of cases. Surgery must therefore be considered the mainstay of OVUF treatment in most patients. Different approaches have been advocated including abdominal transperitoneal transvesical and transvaginal approaches of the fistula. Furthermore, various surgical repair techniques have been developed including conventional open laparoscopy, single-site laparo-endoscopic surgery, robot-assisted surgery [39, 40, 42]. For perimenopausal multiparous women or for those with uterine pathology, hysterectomy is justified [23]. Several authors [23, 43, 44] have reported excellent results after surgical repair of OVUF, reaching 100%.

Surgical repair of the OVUF is most frequently performed via an abdominal approach [23, 43]. According to some authors, to allow direct access to the fistula site and avoid peritoneal contamination, an extraperitoneal transvesical approach appears to be advantageous [45, 46]. However, it is almost impossible to complete the repair without penetrating the peritoneal cavity. For other authors, the transperitoneal route is effective, as it allows satisfactory mobilization of the uterus and the bladder [5, 23]. In our series, we preferred to perform our repairs via an abdominal extra-peritoneal transvesical approach, although in some cases, the transperitoneal transvesical route was required. The success rate of our repairs is encouraging.

This study, which was descriptive, did not establish statistical correlations enabling a deeper understanding of all aspects of VUF in the DRC. We recommend future studies to explore that. However, the present study has the advantage of having reported data covering a wide geographical area (7 provinces in the DRC) and of having a relatively large sample size, on an under-documented subject in DRC. Also, it should be recognized that, only the EUA was used to make the diagnose of OVUFs, which were subsequently confirmed during surgical repair. This is a substantial contribution to the scientific knowledge.

## Conclusion

Obstetric vesico-uterine fistula is relatively common in the DRC, certainly due to the increasing number of caesarean deliveries. Patients are predominantly primiparous, aged between 20 and 29, living in rural areas and with a with limited formal education. Cesarean section is the main cause of this anatomoclinical entity of obstetric urogenital fistula. This fistula is often isolated, single, but its association with ureterovaginal fistula is the most frequent. Fibrosis is absent in a third of cases. Abdominal approach for repair of this fistula is highly effective, with good results in almost all cases. Accessibility to good Cesarean sections and ongoing training for doctors working mainly in rural areas would help reduce the frequency of OVUFs in the DRC.

## Data Availability

The datasets generated and/or analyzed during the current study are not publicly available but are available from the corresponding author on reasonable request.

## Abbreviations

DRC: Democratic Republic of the Congo
OVUF: Obstetric Vesico-uterine Fistula
VUF: Vesico-uterine Fistula
EUA: Examination Under Anesthesia
